# Targeting Human Cancer Cells with Cannabidiol (CBD): Apoptotic Cytotoxicity in HeLa, MDA-MB-231, and CaCo-2 Lines

**DOI:** 10.3390/ijms262412136

**Published:** 2025-12-17

**Authors:** Carlos R. Montes-de-Oca-Saucedo, Jonathan E. Perales-Martínez, Juan C. Arellano-Barrientos, Luis E. Rodríguez-Tovar, Alicia M. Nevárez-Garza, Aimé J. Garza-Arredondo, Odila Saucedo-Cárdenas, Gustavo Hernández-Vidal, Adolfo Soto-Domínguez, Uziel Castillo-Velázquez

**Affiliations:** 1 Departamento de Histología, Facultad de Medicina, Universidad Autónoma de Nuevo León, Monterrey 64460, Mexico; carlos.montess@uanl.edu.mx (C.R.M.-d.-O.-S.); juan.arellanobrr@uanl.edu.mx (J.C.A.-B.); odila.saucedocr@uanl.edu.mx (O.S.-C.); 2 Departamento de Inmunología, Facultad de Medicina Veterinaria y Zootecnia, Universidad Autónoma de Nuevo León, Escobedo 66050, Mexico; jonathan.peralesmrtn@uanl.edu.mx (J.E.P.-M.); luis.rodrigueztv@uanl.edu.mx (L.E.R.-T.); alicia.nevarezgr@uanl.edu.mx (A.M.N.-G.); aime.garzaarr@uanl.edu.mx (A.J.G.-A.); 3 Cuerpo Académico de Patobiología, Facultad de Medicina Veterinaria y Zootecnia, Universidad Autónoma de Nuevo León, Escobedo 66050, Mexico; gustavo.hernandezh@uanl.mx

**Keywords:** cannabidiol (CBD), phytocannabinoids, cytotoxicity, apoptosis, cancer cell lines, LC_50_

## Abstract

Cannabidiol (CBD), a phytocannabinoid derived from *Cannabis sativa*, has demonstrated therapeutic potential across various diseases, including cancer. This study evaluates the cytotoxic effects of CBD on three human cancer cell lines (HeLa, MDA-MB-231, and CaCo-2) and two non-cancerous cell lines (HaCaT and HUVEC) used as a control. Cells were treated with CBD at concentrations of 5, 10, and 20 µM for 24, 48, 72, and 96 h. Cytotoxicity was assessed using MTT assays, nuclear morphology was evaluated via DAPI staining, and cell death mechanisms were analyzed through flow cytometry with apoptosis/necrosis markers. The LC50 values at 24 h were determined as follows: HeLa (9.4 µM), MDA-MB-231 (10.3 µM), and CaCo-2 (4.3 µM). CBD treatment induced morphological changes characteristic of cell stress and death in cancer cells, observed by optical microscopy after 24, 48, 72, and 96 h of exposure. These findings highlight the potential of CBD as an adjunctive therapeutic agent for cancer treatment versus non-malignant cells.

## 1. Introduction

Cancer is one of the leading causes of mortality worldwide, posing a significant burden on public health and healthcare systems. According to the World Health Organization (WHO), over 19.2 million new cases and approximately 9.9 million cancer-related deaths were reported in 2020 [1]. Characterized by uncontrolled cell proliferation and the ability to invade surrounding tissues, cancer presents a highly complex therapeutic challenge. While conventional treatments, including surgery, radiotherapy, and chemotherapy, have demonstrated efficacy in targeting tumor cells, they are often associated with adverse side effects such as anemia, nausea, vomiting, and severe fatigue, significantly compromising patients’ quality of life [2].

Cannabidiol (CBD), a non-psychoactive phytocannabinoid derived from *Cannabis sativa*, has gained attention for its therapeutic potential. It exhibits analgesic, anti-inflammatory, antioxidant, and neuroprotective properties [3,4]. In addition, recent research has suggested that CBD has anticancer potential by inducing molecular mechanisms associated with cell death in breast cancer cells MDA-MB-231, MCF-7, SK-BR-3, and ZR-75-1 [5], as well as in ER-positive, well-differentiated, T-47D and the triple-negative, poorly differentiated, MDA-MB-231 cells. In both cell lines, CBD inhibited cell survival and induced apoptosis dose-dependent observed by MTT assay, morphological changes, DNA fragmentation, and ELISA apoptosis assay [6]. These effects are thought to be mediated through the endocannabinoid system (ECS), a regulatory complex involving cannabinoid receptors (CB1 and CB2), transient receptor potential channels, and lipid ligands [7].

Cannabidiol (CBD) has been reported to possess anticancer potential by inducing molecular mechanisms associated with cell death in various tumor cell types, including breast cancer cells. However, accumulating evidence, including our findings, indicates that these effects are not strictly selective for malignant cells. CBD can also exert measurable cytotoxicity in non-malignant cells, such as keratinocytes (HaCaT) and endothelial cells (HUVEC), particularly at higher concentrations and with prolonged exposure. Therefore, while CBD represents a promising compound with pro-apoptotic activity against cancer cells, its impact on normal cells must also be taken into account to provide a balanced evaluation of its therapeutic applicability.

Despite its promising potential in oncology, CBD’s effects appear to vary depending on cell type, tumor line, concentration, and exposure time [8]. This variability underscores the need for further research to delineate its mechanisms of action and therapeutic applicability. In this study, we evaluated the cytotoxic effects and mechanisms of cell death induced by CBD in three human cancer cell lines (HeLa, MDA-MB-231, and CaCo-2) and two non-cancerous control lines (HaCaT and HUVEC), aiming to explore its potential as an adjunctive therapeutic agent in cancer treatment.

## 2. Results

### 2.1. CBD Induces Morphological Changes in Cancer Cells

The cytotoxic effects and morphological changes induced by CBD were evaluated in HaCaT, HUVEC, HeLa, MDA-MB-231, and CaCo-2 cell lines through optical microscopy. Cells were treated with CBD at concentrations of 5, 10, and 20 μM, as well as controls, for 24, 48, 72, and 96 h. Representative photomicrographs are shown in the corresponding figures. Because cells were already close to confluence at 0 h, vehicle-treated control cultures rapidly entered a plateau phase and therefore showed similar apparent confluence between 0 and 72 h, despite maintaining viability and normal morphology.

**Figure 1:**
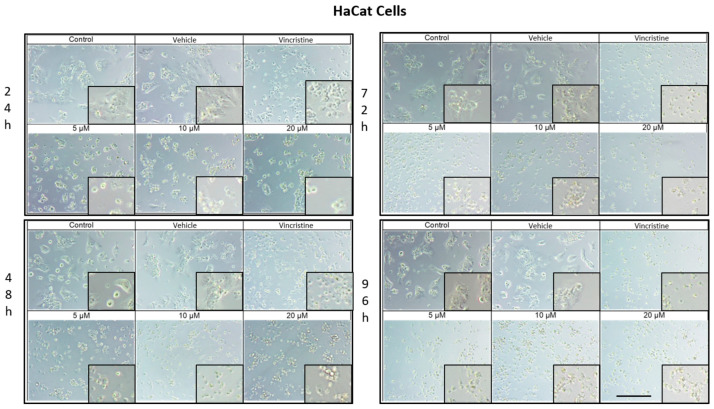
HaCaT cells treated with CBD and controls for 24, 48, 72, and 96 h. Treatments included CBD at concentrations of 5, 10, and 20 μM, 1 μM vincristine, methanol (vehicle), and culture medium (control). Scale bar: 300 μm.

In HaCaT cells treated for 24 h (Figure 1), a reduction in cell confluence was observed at 5 μM, accompanied by morphological alterations such as cell rounding, shrinkage, and loss of adhesion. These effects intensified with higher CBD concentrations (10 and 20 μM), leading to decreased cell confluence and morphological features indicative of stress and cell death. Nevertheless, some cells retained their typical morphology. In comparison, cells treated with vincristine exhibited pronounced changes, including cell rounding, shrinkage, and widespread loss of adhesion.

At 48 h, CBD treatment led to pronounced cytotoxic effects at all tested concentrations, with notable morphological changes associated with stress and cell death. Similarly, vincristine treatment caused cell rounding, shrinkage, poor adhesion, and the presence of cells in suspension.

After 72 h of treatment, HaCaT cells displayed a near-complete loss of adhesion across all CBD concentrations, a characteristic consistent with cell death. Similar effects were observed with vincristine treatment. These findings persisted at 96 h, where cells exposed to CBD and vincristine continued to exhibit the morphological changes observed at earlier time points, suggesting a cumulative cytotoxic effect. In vehicle-treated controls, cells remained confluent from 0 to 72 h, reflecting plateau growth rather than ongoing exponential proliferation.

**Figure 2:**
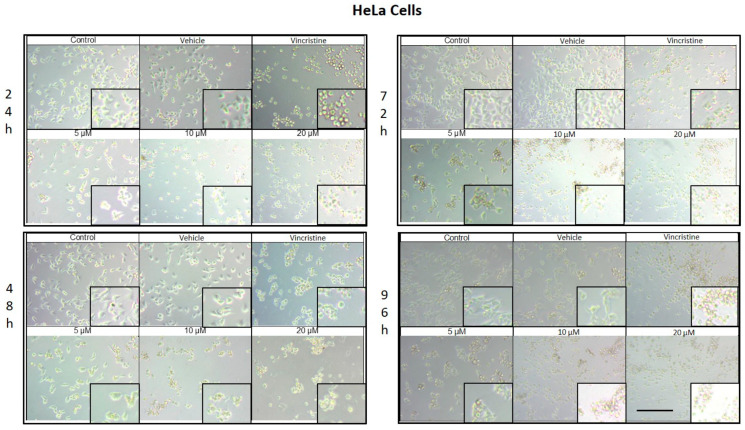
HeLa cells treated with CBD and controls for 24, 48, 72, and 96 h. Treatments included CBD at concentrations of 5, 10, and 20 μM, 1 μM vincristine, methanol (vehicle), and culture medium (control). Scale bar: 300 μm.

CBD treatment induced morphological changes consistent with cellular stress and death in HeLa cells, as observed by optical microscopy at 24, 48, 72, and 96 h of exposure. At 24 h, a reduction in cell confluence was evident at 5 μM, accompanied by cell rounding, shrinkage, and loss of adhesion (Figure 2). These effects were more pronounced at 10 and 20 μM, where nearly all cells exhibited morphologies associated with cell death. Similarly, vincristine treatment caused a marked decrease in confluence and the formation of cell clusters, producing effects comparable to those observed with higher CBD concentrations.

After 48 h of exposure, the cytotoxic effects of CBD intensified. At 10 and 20 μM, most cells were observed in suspension, indicative of severe loss of adhesion. Although some cells treated with 5 μM retained their typical morphology, a noticeable decrease in confluence was observed compared to the controls.

At 72 h, cells exposed to 5 μM of CBD displayed persistent shrinkage, rounding, and cluster formation, whereas treatment with 10 and 20 μM resulted in nearly complete loss of adherence, with cells predominantly in suspension. Vincristine produced similar effects, underscoring the ability of CBD to induce morphological changes characteristic of programmed cell death.

By 96 h, cells treated with 5 μM demonstrated partial resistance, maintaining some cells with typical morphology and showing only a moderate reduction in confluence. However, at 10 and 20 μM, as well as with vincristine, cytotoxic effects remained pronounced, with cells largely in suspension and exhibiting morphologies indicative of cell damage. Similar results in HaCat cells were observed in the HUVEC non-tumor cell line.

**Figure 3:**
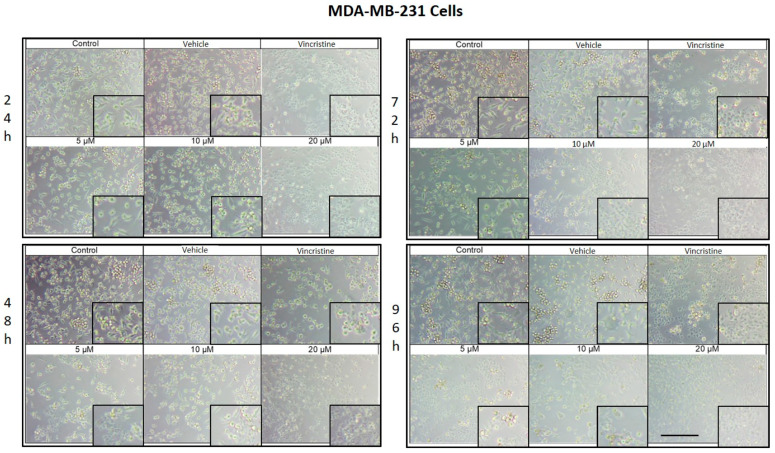
MDA-MB-231 cells treated with CBD and controls for 24 h. Treatments included CBD at concentrations of 5, 10, and 20 μM, 1 μM vincristine, methanol (vehicle), and culture medium (control). Scale bar: 300 μm.

CBD treatment induced morphological changes characteristic of cell stress and death in MDA-MB-231 cells, observed by optical microscopy after 24, 48, 72, and 96 h of exposure. After 24 h, changes such as shrinkage, rounding, and loss of cell adhesion were identified with an increase in the number of cells in suspension, especially starting at 5 μM (Figure 3). Although some cells retained their normal shape, concentrations of 10 μM intensified the effect with more cells in suspension. At 20 μM and with vincristine, the morphological changes were similar, highlighting shrinkage, cell rounding, and the formation of cell clusters associated with stress and cell death.

After 48 h of exposure, the effects of CBD on the MDA-MB-231 line were evident. At 5 μM, cell shrinkage, rounding, cluster formation, and loss of adhesion were observed, accompanied by an increase in cells in suspension. Concentrations of 10 μM produced a similar effect, while 20 μM and vincristine showed effects consistent with those obtained at 24 h, including a significant decrease in cell confluence.

At 72 h, concentrations of 5 and 10 μM demonstrated a decrease in cell confluence, although some cells retained their typical morphology, indicating some resistance to treatment. However, exposures to 20 μM and vincristine resulted in predominantly round, clustered, and suspended cells, reflecting a severe cytotoxic effect.

Finally, after 96 h of treatment, the 5 and 10 μM concentrations showed a decrease in cell confluence, with evidence of stress and cell death, although cells with typical morphology were still observed in a smaller proportion. The 20 μM concentrations and vincristine presented effects similar to those observed at 72 h, with an almost total loss of cell adherence and morphology.

**Figure 4:**
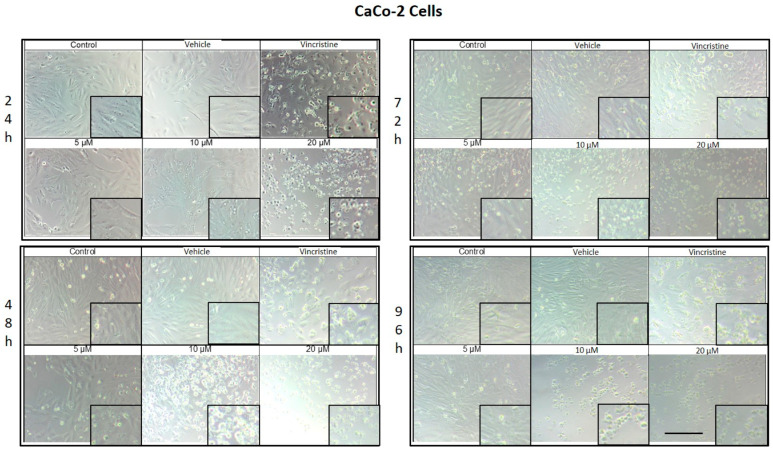
CaCo-2 cells treated with CBD and controls for 24, 48, 72, and 96 h. Treatments included CBD at concentrations of 5, 10, and 20 μM, 1 μM vincristine, methanol (vehicle), and culture medium (control). Scale bar: 300 μm.

CaCo-2 cell cultures exposed to different concentrations of CBD for 24 h demonstrated decreased cell confluence and morphological changes only at 10 and 20 μM, as well as in the positive control with vincristine (Figure 4). At 10 μM, intracytoplasmic vesicles were observed adjacent to the nucleus without significant alterations in overall cell morphology. In contrast, exposure to 20 μM resulted in cell shrinkage, rounding, and loss of adherence, indicative of cellular stress and death. Vincristine induced heterogeneous changes, ranging from light-refracting dead cells in suspension to cells with preserved morphology.

After 48 h, a decrease in cell confluence was observed starting at 5 μM, accompanied by the formation of intracytoplasmic vesicles. At 10 μM, vesicle size increased, and apoptotic bodies and nuclear fragmentation were evident. Rounded cells with loss of adherence were also identified. At 20 μM, the cytotoxic effect was pronounced, with a marked reduction in cell confluence and the presence of suspended debris. Cells treated with vincristine exhibited decreased cell junctions and morphological changes indicative of stress, along with scarce debris in suspension.

By 72 h, cells treated with 5 μM CBD exhibited partial recovery of their typical morphology. However, concentrations of 10 and 20 μM caused shrinkage, rounding, and loss of adherence. Cells exposed to vincristine displayed more aberrant changes, though some cell adherence was retained. Shrinkage and rounding were observed in certain cases.

Finally, after 96 h of exposure, cells treated with 5 μM CBD retained their morphology, suggesting resistance to the treatment. In contrast, at 10 and 20 μM, the cytotoxic effects were evident, with a loss of adherence and morphological changes associated with cellular stress. Vincristine treatment resulted in approximately 60% of cells showing alterations characteristic of stress and cell death.

### 2.2. CBD Decreases Nuclear Fluorescence Intensity in Cancer Cells

Cell viability in CBD-stimulated cells was evaluated by measuring the fluorescence emitted by nuclei stained with the nuclear marker DAPI. This analysis enabled a correlation between fluorescence intensity and the results obtained from the MTT assay.

**Figure 5:**
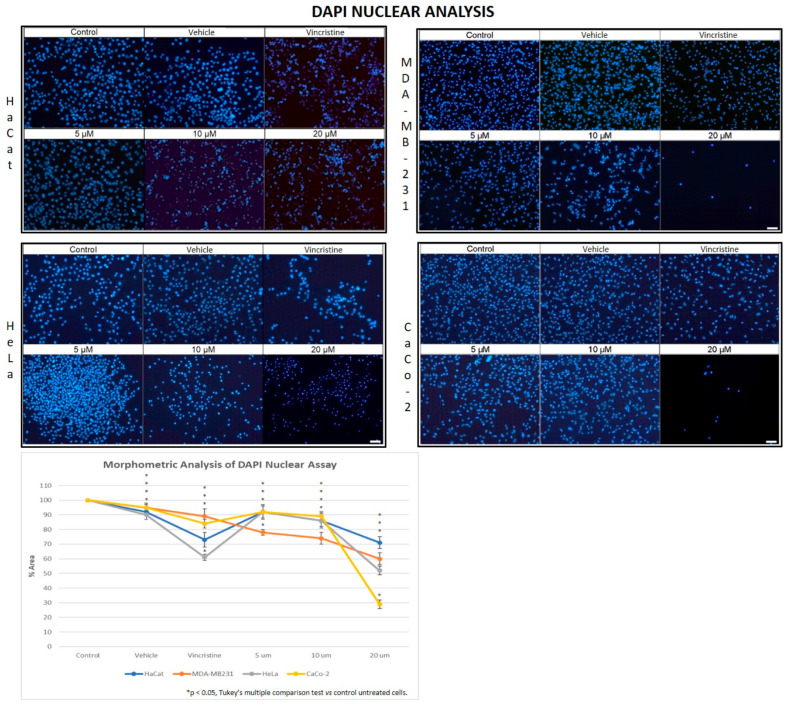
DAPI fluorescence in HaCaT, HeLa, MDA-MB-231, and CaCo-2 cell lines treated for 24 h with different concentrations of CBD and controls. Treatments included 5, 10, and 20 μM of CBD, 1 μM of vincristine (positive control), methanol (vehicle), and culture medium (control). Scale bar: 50 μm. The graph shows the % area as a function of the concentrations of CBD and controls. It can be seen that CBD has a greater cytotoxic effect on the tumor cell lines at 24 h mainly at 20 µm. The CaCo-2 cells showed greater sensitivity to CBD. * *p* < 0.05, Tukey’s multiple comparison test vs. control untreated cells.

In the HaCaT cell line, a progressive decrease in nuclear fluorescence was observed with increasing CBD concentrations after 24 h of exposure (Figure 5). This effect became more pronounced after 48, 72, and 96 h, respectively. At 10 and 20 μM, cells exhibited less defined nuclei with reduced fluorescence intensity. Similarly, cells treated with vincristine showed significant reductions in fluorescence within the first 24 h. These results were similar to those observed in the HUVEC cells.

**Figure 6:**
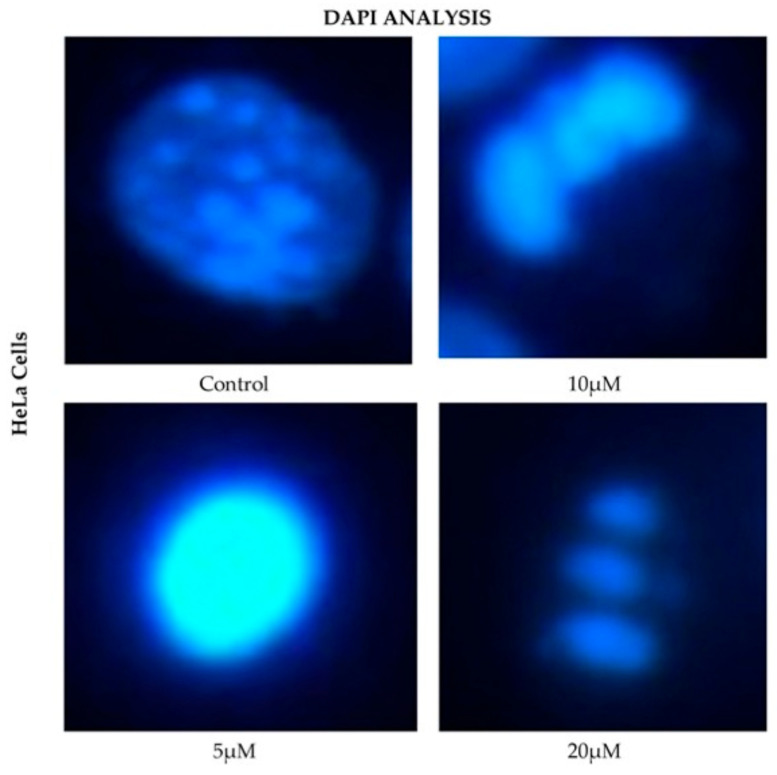
DAPI fluorescence in HeLa cell line treated for 24 h with different concentrations of CBD and controls. Treatments included 5, 10, and 20 μM of CBD and culture medium (control). Scale bar: 10 μm.

HeLa cells displayed a comparable pattern of decreased nuclear fluorescence. At 24 h, cells treated with 5 μM showed a moderate reduction in fluorescence, while 10 and 20 μM treatments resulted in more pronounced attenuation and fragmented nuclei. It is possible to observe reduction in cell volume, marginalization of nuclear chromatin, as well as fragmentation of the nucleus and appearance of apoptotic bodies (Figure 6). These effects were progressively exacerbated at 48, 72, and 96 h, with a clear dose-dependent correlation between CBD concentration and reduced fluorescence intensity.

The response in the MDA-MB-231 cell line was initially more variable. After 24 h, cells exposed to 5 and 10 μM showed a slight reduction in nuclear fluorescence, whereas 20 μM and vincristine resulted in a notable decrease. Over time, these effects intensified, as reflected in Figure 5, where fragmented nuclei and low fluorescence intensity became predominant at higher concentrations.

**Figure 7:**
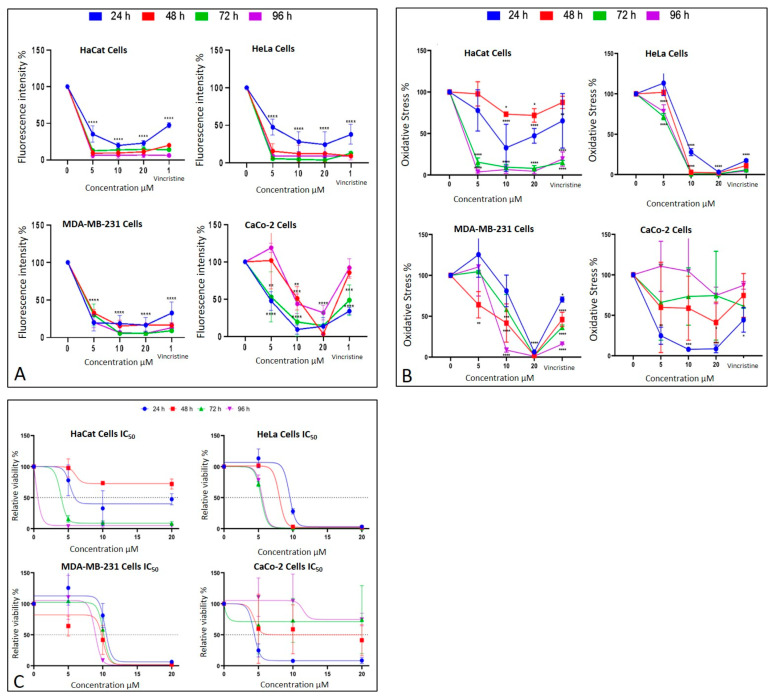
Cell behavior under different CBD concentrations evaluated using the MTT assay. (**A**) Cell viability and (**B**) oxidative stress in HaCaT, HeLa, MDA-MB-231, and CaCo-2 cell lines after 24 h (blue), 48 h (red), 72 h (green), and 96 h (purple) of treatment. Graphs represent the percentage of viable cells and oxidative stress as a function of CBD concentrations. (**C**) Dose–response curves for all evaluated cell lines (HaCaT, HUVEC, HeLa, MDA-MB-231, and CaCo-2), where cell viability (*y*-axis) is plotted as a function of increasing CBD concentrations (0 to 20 µM; *x*-axis). LC_50_ values were determined from these curves for each cell line and exposure time. Results are expressed as mean ± SEM (*n* = 3 independent biological replicates, each performed in triplicate). Statistical analyses were conducted using one-way ANOVA followed by Tukey’s post hoc test against vehicle-treated controls. * *p* < 0.05, ** *p* < 0.01, *** *p* < 0.001, **** *p* < 0.0001.

CaCo-2 cells exhibited a marked initial sensitivity to CBD. At 24 h, treatments with 10 and 20 μM significantly decreased nuclear fluorescence, with nuclei displaying intracytoplasmic vesicles. This effect persisted with longer exposure times (48, 72, and 96 h, where the dose-dependent effects of CBD became evident). Quantitative analyses of fluorescence intensity across all cell lines and exposure times are presented in Figure 7A highlighting cell line-specific responses.

### 2.3. CBD Induces an Alteration of Metabolism in Cancer Cells

The metabolic effects of CBD on cancer cells were assessed using the MTT assay, which quantifies metabolic activity as an indicator of cell viability. The results are presented in Figure 7B corresponding to different exposure times: 24 h, 48 h, 72 h, and 96 h. Each condition was evaluated in three independent biological experiments performed on different days with separate cell cultures, and within each experiment, all treatments were analyzed in technical triplicates. Thus, each data point represents the mean of nine measurements (3 biological × 3 technical replicates).

After 24 h of exposure, HeLa cells exhibited a significant decrease in viability starting at 10 μM, reflecting high sensitivity to CBD. MDA-MB-231 cells showed a reduction in viability only at the highest concentration (20 μM), indicating initial resistance. CaCo-2 cells, in contrast, displayed significant sensitivity across all tested concentrations, while HaCaT and HUVEC cells demonstrated a moderate response without notable changes in viability.

At 48 h, HeLa cells maintained their marked sensitivity, with significant reductions in viability at all concentrations except 5 μM. MDA-MB-231 cells began to show a concentration-dependent cytotoxic response, with viability decreasing significantly at 10 and 20 μM. Interestingly, CaCo-2 cells displayed emerging resistance, with smaller reductions in viability compared to earlier exposure times. HaCaT cells continued to exhibit minimal sensitivity during this period.

By 72 h, HeLa cells retained their high sensitivity, showing pronounced viability reductions even at lower concentrations of CBD. MDA-MB-231 cells demonstrated limited sensitivity, with significant viability reductions observed only at 20 μM. CaCo-2 cells showed sustained resistance, suggesting possible adaptive mechanisms. HaCaT cells, however, began to display significant sensitivity, particularly at 10 and 20 μM.

Finally, after 96 h of exposure, HeLa cells continued to exhibit high sensitivity, with significant reductions in viability across all concentrations. MDA-MB-231 cells showed increased sensitivity at 10 and 20 μM, indicating a cumulative cytotoxic effect. CaCo-2 cells maintained their resistance, with higher viability compared to the other cell lines. HaCaT and HUVEC cells, despite being non-cancerous, demonstrated a marked decrease in viability, indicating a cytotoxic effect associated with prolonged exposure and higher doses.

These findings emphasize the variability in the response of different cell lines to CBD. HeLa and CaCo-2 cells were most sensitive to treatment during early stages, whereas MDA-MB-231 and HaCaT cells exhibited relative resistance, which evolved over longer exposure times. This differential sensitivity underscores the importance of considering the unique characteristics of each cell type when evaluating the therapeutic potential of CBD.

### 2.4. CBD Decreases Cell Viability in Cancer Cells

The MTT assay was used to assess cell viability in HaCaT, HUVEC, HeLa, MDA-MB-231, and CaCo-2 cell lines following exposure to various concentrations of CBD (5, 10, and 20 μM) and controls for 24, 48, 72, and 96 h. The results, presented in Figure 7B, underscore the differential sensitivity of the cell lines and the dose- and time-dependent effects of CBD.

In HeLa and HUVEC cells, a significant reduction in cell viability was observed starting at 10 μM after 24 h of exposure. This effect persisted and intensified over time, with more pronounced decreases at 72 and 96 h. In contrast, MDA-MB-231 cells exhibited greater initial resistance, with a notable reduction in viability observed only at 20 μM during the first 24 h. However, prolonged exposure (72 and 96 h) increased their susceptibility, confirming a dose- and time-dependent response.

CaCo-2 cells demonstrated high initial sensitivity, with significant viability reductions even at the lowest concentration (5 μM) after 24 h. Interestingly, these cells exhibited progressive resistance during longer exposures, particularly at 72 and 96 h. This shift suggests the activation of adaptive mechanisms to counteract the cytotoxic effects of CBD.

HaCaT and HUVEC cells, serving as a non-cancerous control, showed minimal sensitivity to CBD during shorter exposures. However, at 72 and 96 h, a substantial decrease in viability was observed, particularly at 10 and 20 μM, highlighting the potential for long-term effects of CBD even in non-malignant cells.

These findings emphasize the heterogeneity in the cellular responses to CBD, which can be attributed to intrinsic differences in metabolism and resistance mechanisms among cell lines. While the results support the potential of CBD as a selective therapeutic agent, they also stress the necessity of optimizing treatment parameters, such as dose and exposure duration, tailored to the characteristics of the target cells (Figure 7).

### 2.5. Determination of the Median Inhibitory Dose (LC_50_) of CBD in Cancer Cell Lines

The MTT assay results facilitated the calculation of the LC_50_ values for each cell line at different exposure times (Figure 7C). These values highlight the varying sensitivity of the tested cell lines to CBD over time.

HaCaT cells, and the HUVEC line as non-cancerous lines, demonstrated progressive sensitivity to CBD, with LC_50_ values decreasing significantly as exposure times increased, reaching their lowest value (0.183 μM) for the HaCat line and (0.268 μM) for the HUVEC line at 96 h. This indicates a cumulative cytotoxic effect of CBD on this line with prolonged treatment. HeLa cells exhibited high sensitivity to CBD, with an initial LC_50_ of 9.495 μM at 24 h, which further decreased to approximately 5.5 μM at longer exposure times. This trend suggests that the susceptibility of HeLa cells to CBD intensified with extended exposure. MDA-MB-231 cells, characterized by their intrinsic resistance to various treatments, presented relatively stable LC_50_ values, ranging between 9 and 10 μM across all exposure times. This stability reflects a resistance mechanism that remains consistent despite prolonged CBD exposure.

In contrast, CaCo-2 cells were the most sensitive at shorter times, with an initial LC_50_ of 4.34 μM at 24 h. However, a slight increase in LC_50_ values at extended exposure times suggests the activation of adaptive resistance mechanisms within this cell line (Figure 7C).

An important anomaly was observed in the LC_50_ values, as HaCaT cells showed an exceptionally low LC_50_ at 96 h (0.183 µM), which is markedly lower than both earlier time points and the values reported for malignant cell lines. After re-examining the raw data and repeating the experiments, this trend was confirmed, suggesting that prolonged exposure leads to a cumulative effect. We propose that this drastic reduction in LC_50_ may be associated with progressive cellular stress, depletion of defense mechanisms, or potential CBD accumulation over time. Previous studies have reported much higher IC_50_ values for HaCaT under shorter exposures or different contexts. For instance, Li et al. reported an IC_50_ ≈ 20.87 µM in HaCaT after 24 h under UVB stress [9]; Mazzantini et al. found no significant toxicity at 0.001–1 µM after 24 h exposure [10]; and Casares et al. identified that CBD activates antioxidant pathways through Nrf2 and BACH1 modulation in keratinocytes [11]. Taken together, these comparisons suggest that the very low LC_50_ we report at 96 h may reflect a time-dependent cumulative sensitivity in HaCaT cells rather than an experimental error. This finding highlights the importance of considering prolonged exposure effects when interpreting CBD cytotoxicity and its selectivity profile.

The summarized data in Table 1 reinforce these findings, emphasizing the differential responses of each cell line to CBD and the importance of considering both time and cell type when evaluating CBD’s therapeutic potential.

### 2.6. Type of Cell Death Induced by CBD

**Figure 8:**
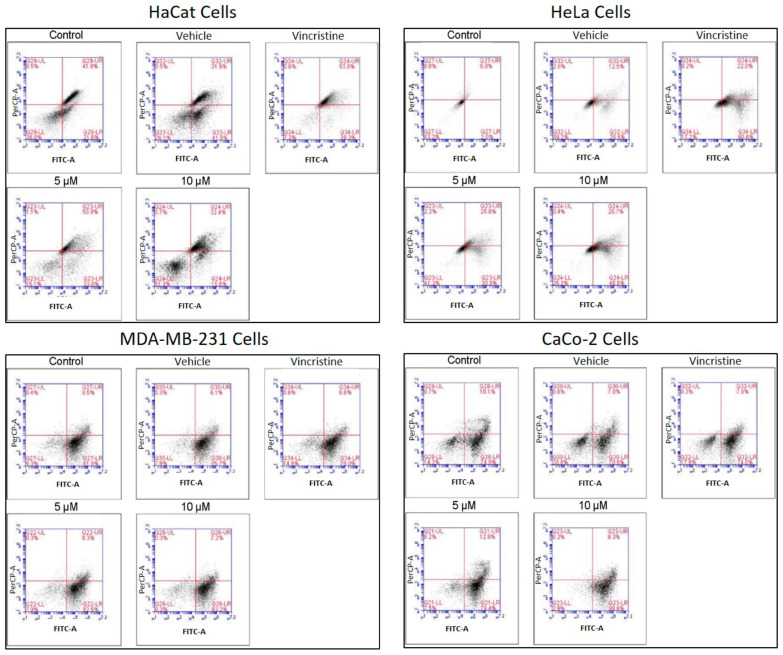
Flow cytometry analysis of cell death types in HaCaT, HeLa, MDA-MB-231, and CaCo-2 cells treated with CBD and controls for 24 h. Cells were stained with 7-AAD and Apopxin Green to differentiate stages of cell death. Each large panel corresponds to a specific cell line: HaCaT, HeLa, MDA-MB-231, and CaCo-2. Within each panel, dot plots represent the treatment conditions: control, vehicle (methanol), vincristine (positive control), 5 μM CBD, and 10 μM CBD. In each dot plot, the quadrants are defined as follows: the lower-left quadrant shows viable cells, the lower-right quadrant shows cells in early apoptosis, the upper-right quadrant corresponds to late apoptosis, and the upper-left quadrant indicates necrotic cells.

The type of cell death induced by CBD was evaluated using a commercial apoptosis/necrosis detection kit (Abcam, cat. ab176749, Cambridge, MA, USA) and analyzed through flow cytometry. Representative dot plots for the HaCaT, HeLa, MDA-MB-231, and CaCo-2 cell lines are displayed in Figure 8.

Flow cytometry analysis, performed in three independent experiments (≥10,000 events per sample) and reanalyzed in FlowJo using a stringent and standardized gating strategy, revealed a clear cell line–dependent response to the treatment. In HeLa cells, viability decreased from 90.99% (control) and 84.3% (vehicle) to 63.83% and 67.37% in the presence of 5 and 10 µM of the compound, respectively, with a concomitant increase in total apoptosis (early + late) from 9 to 15% up to approximately 33–36%, although still lower than the positive control (56%). In contrast, MDA-MB-231 and CaCo-2 cells maintained high viability (≈81–88%) under all treatments, and only modest changes in total apoptosis (≈9–17%) were observed, even in the positive control, indicating a marked intrinsic resistance. After reanalysis, HaCaT keratinocytes emerged as the most sensitive cell line: control and vehicle samples showed high viability with only basal apoptosis, whereas treatment with 5 µM of the compound markedly reduced viability and strongly increased total apoptosis to values approaching those of the vincristine-treated positive control, while 10 µM produced a more moderate pro-apoptotic effect. Overall, these data indicate that the compound predominantly induces apoptotic rather than necrotic cell death and exhibits strong pro-apoptotic activity in HaCaT cells, moderate effects in HeLa cells and minimal impact on MDA-MB-231 and CaCo-2 cells.

In HaCaT cells, CBD primarily induced early and late apoptosis, with minimal levels of necrosis even at the highest concentrations tested. This pattern indicates a preference for programmed cell death pathways in non-cancerous cells under CBD treatment. HeLa cells exhibited a similar response, with significant increases in both early and late apoptosis observed from 5 μM. The apoptotic effect intensified with higher concentrations, while necrosis levels remained low, reinforcing the pro-apoptotic activity of CBD in this cell line. In MDA-MB-231 cells, the response to CBD was more heterogeneous. Initial resistance was observed at lower concentrations, with a notable transition to late apoptosis at higher concentrations, indicating a concentration-dependent apoptotic effect in this triple-negative breast cancer cell line. CaCo-2 cells demonstrated high sensitivity to CBD, with a predominance of early and late apoptosis across all concentrations tested. This response highlights the susceptibility of this colorectal cancer cell line to CBD-induced programmed cell death.

The quantitative analysis of cell viability, apoptosis, and necrosis for each treatment is summarized in Table 2. These findings underscore the ability of CBD to selectively induce apoptosis in cancer cell lines while maintaining minimal necrotic activity, supporting its potential as a therapeutic agent. It should be noted that untreated control cultures remained confluent and morphologically preserved in phase-contrast microscopy, and retained high metabolic activity in the MTT assay together with normal nuclear morphology and fluorescence in DAPI staining. Therefore, the relatively high baseline fractions of cells classified as ‘apoptotic’, particularly in MDA-MB-231 and CaCo-2 controls, likely reflect the sensitivity of the Apopxin Green/7-AAD assay to early membrane and nuclear alterations rather than a loss of over-all culture viability.

Table 2 Apoptotic cell death induced by CBD in HaCaT, HUVEC, HeLa, and MDA-MB-231 cell lines at 5 and 10 µM, evaluated by flow cytometry after 48 h of treatment. Data are expressed as mean ± SEM (*n* = 3 independent biological replicates, each performed in triplicate). Statistical comparisons were performed using one-way ANOVA followed by Tukey’s post hoc test versus vehicle-treated controls. *p* < 0.05 was considered statistically significant.

## 3. Discussion

The findings of this study confirm the potential of CBD as a cytotoxic agent with pro-apoptotic activity in cancer cells. While previous research has demonstrated that CBD can induce apoptosis and cellular stress in various cancer types, including cervical, breast, and colorectal adenocarcinomas, our results also show that its effects are not strictly selective for malignant cells. In particular, HaCaT cells exhibited significant apoptosis (51.05% at 5 µM), indicating that non-malignant cells can also be affected under certain conditions [5,6]. These observations highlight the need for further studies to elucidate the mechanisms underlying this differential sensitivity. The relatively high basal apoptotic fractions observed in some control conditions, especially in MDA-MB-231 and CaCo-2, should thus be interpreted in conjunction with MTT and DAPI data and regarded as a limitation of the apoptosis/necrosis assay rather than as evidence of a methodological error.

CBD appears to exert a multifaceted mechanism of action on tumor cells, involving both the endocannabinoid system and receptor-independent pathways. It can interact with CB1 and CB2 receptors, but antitumor effects have also been reported in models where inhibition of tumor cell migration occurs independently of these receptors [12]. At the cellular level, CBD has been shown to induce apoptosis, autophagy and cell-cycle arrest, and to inhibit tumor cell invasion and angiogenesis [13]. In addition, CBD can reduce inducible nitric oxide synthase (iNOS) expression and nitric oxide (NO) production via CB1 activation [14], thereby modulating the inflammatory tumor microenvironment and potentially contributing to the cytotoxic and pro-apoptotic effects observed in the present study [13].

Beyond its pro-apoptotic effects, CBD also modulates cellular redox balance in a context-dependent manner. In non-malignant models, such as human umbilical vein endothelial cells (HUVECs) and human polymorphonuclear leukocytes, CBD pre-treatment has been shown to reduce ROS and malondialdehyde (MDA) levels, enhance antioxidant enzyme activities and dampen the oxidative burst, consistent with a protective antioxidant profile [15,16]. By contrast, in glioblastoma cells CBD can increase intracellular ROS via ERK activation, thereby promoting autophagy and ferroptosis and contributing to tumor cell death [17]. Recent reviews emphasize that these dual antioxidant and pro-oxidant actions depend on cell type, dose, exposure time and pathological context, and that CBD can regulate oxidative signaling at multiple levels [18,19]. Although ROS were not directly measured in the present study, CBD-induced modulation of oxidative stress may partially account for the cytotoxic and pro-apoptotic effects observed here, particularly in the most sensitive cell lines.

Interestingly, our findings in HaCaT keratinocytes contrast with reports by Li et al., who suggested that CBD could protect HaCaT cells through autophagy activation. This discrepancy may be explained by differences in CBD concentration, exposure time, or experimental microenvironment. While Li et al. observed that autophagy promoted cell survival, our prolonged exposure and higher concentrations appear to favor pro-apoptotic pathways, likely mediated by oxidative stress and mitochondrial dysfunction [9]. These results suggest that autophagy may act as a double-edged sword, providing cytoprotection under certain conditions but shifting toward apoptosis when oxidative stress becomes dominant.

Morphological changes observed under optical microscopy included membrane contraction, cell rounding, loss of adhesion, vesicle formation, and surface protrusions—hallmarks of cellular stress and apoptosis. These findings corroborate earlier reports in HeLa [20] and MDA-MB-231 cells [6]. For CaCo-2 cells, although CBD-induced cytotoxicity has been documented [21,22], this is the first report describing such detailed morphological changes. Similarly, HaCaT cells exhibited stress and death-related alterations, but some retained their typical morphology, a phenomenon not previously reported in the literature [9,23,24].

The MTT assay revealed variability in CBD sensitivity across the cell lines, as evidenced by differences in LC_50_ values. CaCo-2 cells demonstrated the highest sensitivity, with an LC_50_ of 4.34 μM at 24 h, which is lower than previously reported values [21,22]. MDA-MB-231 cells exhibited an LC_50_ of 10.37 μM, higher than reported in other studies (2.2 μM), likely due to differences in experimental conditions [6]. HeLa cells had an LC_50_ of 9.49 μM, consistent with findings from Lukhele and Motadi (2016) [25]. Interestingly, HaCaT cells showed significant cytotoxicity at higher CBD concentrations, with an LC_50_ of 5.23 μM, diverging from earlier studies that reported minimal effects at similar concentrations [23]. It should also be noted that tetrazolium-based assays such as MTT may be prone to interference by plant-derived compounds, including cannabinoids, which can directly reduce MTT and potentially lead to an overestimation of cell viability [26].

Nuclear changes detected by DAPI staining, such as chromatin condensation, nuclear fragmentation, and apoptotic body formation, are characteristic of apoptosis and correlated with reduced fluorescence intensity, reflecting diminished cell viability [27,28]. Similar observations were made in cancer cell lines HeLa [25], MDA-MB-231 [29], and CaCo-2 [30], while in HaCaT cells, such changes had not been previously reported in the context of CBD treatment [9,23,24].

The apoptosis/necrosis assay confirmed that CBD predominantly induces apoptosis across all cell lines. In HeLa cells, early apoptosis reached 52.8% at 10 μM, consistent with previous studies [25]. In MDA-MB-231 cells, apoptosis rates were high (82.6% at 5 μM and 81.7% at 10 μM), although not dose-dependent, possibly due to mechanisms like autophagy or bubbling cell death [5,29]. CaCo-2 cells showed remarkable sensitivity, with 79.1% apoptosis at 5 μM and 87.0% at 10 μM, even at lower concentrations than previously evaluated [30]. HaCaT cells also showed significant levels of apoptosis, but the results differed from earlier findings [24], likely due to experimental variations. It is important to note that apoptosis was evaluated only in adherent cells, so apoptotic events in detached cells may be underestimated.

In HaCaT cells, the molecular mechanisms underlying CBD-induced cytotoxicity remain unclear. Evidence suggests that CBD may increase cytosolic calcium levels through activation of TRPV1 receptors [31], which facilitates calcium flux into mitochondria via VDAC1 receptors. This process could lead to mitochondrial permeability transition pore formation, mitochondrial membrane depolarization, dysfunction, and ultimately, apoptosis [32].

Under stress conditions, such as oxidative stress, autophagy often plays a protective role in maintaining cell homeostasis, for example, CBD induces melanoma cell death by initiating cell autophagy, so it is difficult to observe a direct selectivity between tumor cells and non-malignant cells so that we can consider autophagy as a double-edged sword for cell survival and cell death [33]. Some studies reported the role of CBD in inhibiting NF-κB and in inducing autophagy to protect non-tumor cells, such as SH-SY5Y, from mitochondrial dysfunction [34]. On the other hand, in HUVEC cells CBD induced cytostasis without inducing apoptosis, inhibited migration, invasion, and sprouting of umbilical cells in vitro, and in vivo in matrigel sponges.

These results underscore the potential of CBD as an adjunctive therapy in cancer treatment, particularly for colorectal adenocarcinoma, where it exhibited greater efficacy. However, the variability in responses among cell lines highlights the importance of tailoring CBD-based treatments to the molecular profile of each cancer type. Combining CBD with chemotherapeutic agents could further enhance its efficacy, particularly in resistant lines like MDA-MB-231.

Previous studies have also investigated the effects of CBD on non-malignant HaCaT keratinocytes. Talebi et al. reported that CBD modulates oxidative stress responses by activating Nrf2 and autophagy pathways under UVB exposure, indicating that non-cancerous cells are sensitive to CBD-induced stress [35]. Similarly, Fumagalli et al. demonstrated that a standardized Cannabis sativa extract containing ~5% CBD did not display cytotoxicity in HaCaT cells under pro-inflammatory conditions up to certain concentrations, suggesting that toxicity strongly depends on dose and exposure context [36]. In addition, a recent review by Nahler et al. highlighted that although phytocannabinoids combined with chemotherapeutic agents have shown promise in preclinical cancer models, the evidence remains heterogeneous and scarce regarding their safety in non-malignant cell lines such as HaCaT [37]. These observations reinforce the importance of considering both malignant and non-malignant models when evaluating the therapeutic potential and limitations of CBD.

Combining CBD with chemotherapeutic agents could further enhance its efficacy, particularly in resistant lines like MDA-MB-231. Evidence from preclinical studies supports this rationale: Go et al. demonstrated that CBD enhanced the antitumor activity of cisplatin in head and neck squamous cell carcinoma models [38]; Chen et al. reported that natural and synthetic cannabinoids potentiated the cytotoxicity of cisplatin in multiple cancer cell lines [39]; and Ismail et al. showed that in ovarian cancer cells, CBD combined with cisplatin or paclitaxel produced either antagonistic or synergistic effects depending on the treatment regimen [40]. These findings indicate that while CBD may improve chemotherapy efficacy under certain conditions, treatment timing and dosing are critical factors that warrant further investigation.

This study’s main limitation is the exclusive use of in vitro models, which limits direct extrapolation to clinical settings. Future research should focus on validating these findings in vivo models, investigating CBD’s synergistic effects with existing chemotherapeutic agents, and evaluating its activity in complex tumor microenvironments.

## 4. Material and Methods

### 4.1. CBD Source and Chemical Characterization

The CBD employed in this study (1 g, H87, Lot 2347027) was purchased from Farmacias de Especialidades Dermatológicas de México (FA-DERMEX.com, Ecatepec de Morelos, Mexico). According to the certificate of analysis (COA), it had a purity > 99% with no detectable THC or contaminants (Table 3). For experimental use, CBD was weighed as a pure powder, dissolved in methanol to prepare a stock solution, and subsequently diluted in DMEM to obtain the desired working concentrations. Vehicle controls received the same final concentration of methanol as CBD-treated wells, while negative controls received culture medium only. According to the certificate of analysis (COA) given by the company, chemical characterization analysis revealed the following composition (Table 3).

### 4.2. Cell Culture

Five cell lines were used: HeLa (ATCC: CCL-2), MDA-MB-231 (ATCC: HTB-26), CaCo-2 (ATCC: HTB-37), HUVEC (ATCC: CRL-1730), and HaCaT (Cat. No. 300493). HeLa cells, derived from human cervical adenocarcinoma, are characterized by rapid proliferation, low p53 expression, and overexpression of cannabidiol receptors. MDA-MB-231 cells, isolated from triple-negative mammary adenocarcinoma, are highly invasive and resistant, with mutations in *KRAS* and *TP53*. CaCo-2 cells, derived from colorectal adenocarcinoma, exhibit mutations in *APC*, β-catenin, and *TP53*, along with overexpression of COX2. HUVEC is an endothelial cell line isolated from the vein of the umbilical cord, which overexpresses vascular endothelial growth factor (VEGF). HaCaT cells, immortalized human keratinocytes, were also included as a non-malignant control for cytotoxicity evaluation.

HeLa, MDA-MB-231, CaCo-2, and HUVEC cell lines were purchased from the American Type Culture Collection (ATCC, Manassas, VA, USA). HaCat cell line was obtained from Cytion (Eppelheim, Germany). All cell lines were cultured in Dulbecco’s Modified Eagle Medium (DMEM) supplemented with 10% inactivated fetal bovine serum, 1% penicillin-streptomycin, and 1% glutamine. Cells were incubated at 37 °C in a humidified atmosphere containing 5% CO_2_. Media was refreshed every two days until cells reached 80% confluence, at which point the experiment tests were initiated.

### 4.3. Cell Viability Assay

Cellular metabolic activity was assessed using the MTT assay (3-[4,5-dimethylthiazol-2-yl]-2,5-diphenyltetrazolium bromide), which measures the reduction in tetrazolium to formazan as an indicator of cell viability. Cells were seeded in 96-well plates at a density of 1 × 10^4^ cells per well, resulting in approximately 70–80% confluence at the time defined as 0 h, and then treated with CBD at concentrations of 5, 10, and 20 µM for 24, 48, 72, and 96 h. MTT assays were performed in three independent biological experiments conducted on different days with separate cell cultures, each carried out in triplicate. Thus, a total of nine values were obtained for each condition. Control groups included untreated cells (negative control), cells treated with 1 µM vincristine (positive control), and cells exposed to methanol (vehicle control). After incubation, MTT solution (0.5 mg/mL) was added and incubated for 4 h at 37 °C. The CBD used in this study (FADERMEX.com, Morelia, Mexico) was characterized in our laboratory by LC-MS/MS and GC-MS/MS to confirm composition and purity (>99%) and to exclude THC. Additionally, ICP-MS was employed for heavy metals, and screening for pesticides, solvent residues, molds, and fungi (SOP 202 and SOP 301) was performed, with no contaminants detected. The resulting formazan crystals were dissolved in DMSO, and absorbance was measured at 570 nm using a microplate reader. Results were expressed as the percentage of cell viability relative to the negative control, with significant cytotoxicity defined as a ≥30% reduction in cell viability, as per ISO-10993-5-2009 guidelines [41].

### 4.4. Nuclear Fragmentation Assay

To investigate nuclear alterations associated with apoptosis, cells were stained with DAPI (4′,6-diamidino-2-phenylindole), which binds specifically to DNA and highlights nuclear morphology [42]. Cells were fixed with 4% paraformaldehyde for 15 min at room temperature and permeabilized with 0.1% Triton X-100 for 10 min. After staining with DAPI (1 μg/mL) for 15 min in darkness, nuclei were visualized using fluorescence microscopy equipped with DAPI-specific filters. Nuclear changes characteristic of apoptosis, such as chromatin condensation and nuclear fragmentation, were analyzed and correlated with cell viability. While these nuclear alterations suggest apoptotic processes, the formation of apoptotic bodies was inferred based on nuclear morphology and further corroborated using complementary flow cytometry assays described below.

For the DAPI nuclear morphometric assay six micrographs were taken per concentration at a total magnification of 100×, which were evaluated with ImageJ software version 1.51, (NIH) which calculated the percentage of the area covered by the adhered cells in each of the wells [43].

### 4.5. Apoptosis and Necrosis Assay

To complement the analysis of nuclear alterations, cell death mechanisms induced by CBD were assessed using the Apopxin Green/7-AAD kit (Abcam, cat. ab176749, Cambridge, MA, USA). This assay differentiates between early apoptosis, late apoptosis, and necrosis. Treated cells were harvested via gentle trypsinization, washed with cold PBS, and resuspended in binding buffer. Staining was performed according to the manufacturer’s instructions. Samples were analyzed using a BD Accuri C6 Plus flow cytometer (BD, Ashland, OR, USA), collecting at least 10,000 events per sample. The resulting data provided quantitative insights into the distribution of cells in various stages of apoptosis and necrosis, enabling a comprehensive interpretation of the mechanisms underlying reduced cell viability. For each experiment, all treatment groups (control, vehicle, positive control, and CBD) were seeded at the same initial cell density and were stained and acquired in parallel under identical instrument settings, collecting at least 10,000 events per sample. Gates were created for each independent cell lines.

Flow cytometry data (.fcs files) were analyzed using FlowJo v11 software (FlowJo LLC, BD Biosciences Ashland, OR, USA). A sequential gating strategy was applied: first, an FSC-A vs. SSC-A gate was used to exclude debris and select the main cell population, followed by doublet exclusion using FSC-H vs. FSC-A to retain singlets only. Within singlets, viable cells were identified based on CytoCalcein Violet 450 and 7-AAD staining and then analyzed on Apopxin Green (FITC) vs. 7-AAD dot plots. Quadrants were set using unstained, single-stained and fluorescence-minus-one controls to define positivity thresholds. For each condition, the percentage of cells in each population (viable, early apoptotic, late apoptotic and necrotic) and the median fluorescence intensity (MFI), percentage of positive events and fluorescence peak shifts were quantified in FlowJo.

### 4.6. Statistical Analysis

All experiments were conducted in three independent biological replicates, each performed in triplicate. Data distribution was tested for normality using the Shapiro–Wilk test and for homoscedasticity using Levene’s test prior to applying one-way ANOVA. When significant differences were detected, Tukey’s post hoc test was used for multiple comparisons against vehicle-treated controls. Results are expressed as mean ± SEM, with significance set at *p* < 0.05. Exact *p*-values and 95% confidence intervals are reported. Although formal effect size calculations (e.g., Cohen’s *d*, η^2^) were not included, reproducibility across biological replicates supports the robustness of the observed differences. Logarithmic graphs and median inhibitory concentrations (LC_50_) were determined using GraphPad Prism 9 software.

## 5. Conclusions

In the present study, we demonstrated that CBD induces apoptosis in human cancer cells (HeLa, MDA-MB-231, and CaCo-2) while delaying apoptotic processes in non-malignant control cells (HaCaT and HUVEC). These findings underscore the potential of CBD as an adjunctive therapeutic agent for cancer treatment, highlighting its selective cytotoxic effects on malignant cells with limited impact on non-malignant cells.

Understanding that the exclusive use of CBD would not be enough to treat or eliminate cancer cells, the combination with agents or in antitumor therapies can be an excel-lent adjuvant and show a synergistic action, likewise, it has clinical benefits such as anti-inflammatory, antioxidant, antiemetic, appetite stimulant activity that can be favorable in the control of such signs associated with chemotherapies, improving the quality of life and adverse effects of these antitumor therapies.

Future studies should focus on optimizing CBD dosing and evaluating its efficacy in combination with conventional therapies to maximize its therapeutic potential in clinical oncology. Also, further studies evaluating the effects of CBD over other non-tumor cell lines like HEK-293, 3T3 fibroblast, Vero cells, etc., should be performed.

## Figures and Tables

**Table 1:** Determination of mean inhibitory dose LC_50_ per cell line under CBD exposition.

Cell Line	Time of Exposure	LC_50_	SEM
HaCaT	24 h	5.233	0.417
	72 h	3.903	0.157
	96 h	0.183	1.525
HUVEC	24 h	2.1647	0.419
	48 h	2.6887	0.265
	72 h	1.3471	0.256
	96 h	0.268	0.058
HeLa	24 h	9.495	0.157
	48 h	8.034	0.555
	72 h	5.400	0.029
	96 h	5.547	0.074
MDA-MB-231	24 h	10.373	0.248
	48 h	9.993	0.292
	72 h	10.110	0.105
	96 h	8.893	0.848
CaCo-2	24 h	4.340	0.122
	48 h	4.389	1.299

**Table 2:** Type of cell death induced by 5 and 10 µM CBD and controls in cell lines after 24 h of exposure, analyzed by flow cytometry.

Flow Cytometry Analysis					
Cell Line	Concentration	Viability (%)	Early Apoptosis (%)	Late Apoptosis (%)	Necrosis (%)
HaCaT	Control	80.00	6.47	12.53	1.00
	Vehicle	85.00	3.84	10.66	0.50
	Vincristine	10.00	37.09	51.91	1.00
	5 µM	20.00	30.68	47.32	2.00
	10 µM	70.00	9.43	19.57	1.00
HeLa	Control	91.00	2.70	6.00	0.30
	Vehicle	84.30	2.80	12.50	0.40
	Vincristine	42.00	34.00	22.00	2.00
	5 µM	63.48	10.65	25.47	0.40
	10 µM	67.33	6.59	25.67	0.41
MDA-MB-231	Control	81.10	8.60	8.50	1.80
	Vehicle	86.30	6.30	6.10	1.30
	Vincristine	77.80	10.60	6.60	5.00
	5 µM	85.00	6.00	7.30	1.70
	10 µM	85.50	6.11	7.10	1.29
CaCo-2	Control	84.52	2.04	9.83	3.61
	Vehicle	87.91	1.00	7.79	3.30
	Vincristine	82.25	6.37	7.93	3.45
	5 µM	82.80	2.00	12.90	2.30
	10 µM	86.30	0.60	8.30	4.80

**Table 3:** Phytocannabinoid Content of CBD Isolate (Analyzed by Dry Weight).

Abbreviation	Dry Wt. %	Dry Wt. mg/g
THCA	<LOQ	<LOQ
Δ-9-THC	<LOQ	<LOQ
Δ-8-THC	<LOQ	<LOQ
THCV	<LOQ	<LOQ
CBDA	<LOQ	<LOQ
CBD	99% (A)	990.0 mg/g
CBGA	<LOQ	<LOQ
CBG	<LOQ	<LOQ
CBDVA	<LOQ	<LOQ
CBDV	1.00%	10.0 mg/g
CBN	<LOQ	<LOQ
CBL	<LOQ	<LOQ
CBC	<LOQ	<LOQ

## Data Availability

The original contributions presented in this study are included in the article. Further inquiries can be directed to the corresponding authors.
